# Complication of Geniculate Artery Embolization

**DOI:** 10.1016/j.acepjo.2025.100249

**Published:** 2025-09-08

**Authors:** Ziomara Jurado, Dhara P. Amin

**Affiliations:** Department of Medicine, Section of Emergency Medicine, University of Chicago, Chicago, Illinois, USA

**Keywords:** complication, safety, procedures

## 1 Patient Presentation

A 45-year-old woman with a past medical history of osteoarthritis to the bilateral knees presented to the emergency department with pain that was lateral to the ball of her foot, a subjective cool feeling to her toes, and swelling to her left knee for 1 day. The patient had a geniculate artery embolization with interventional radiology the day prior to presentation. Physical examination was notable for an intact palpable and Doppler-able posterior tibial artery and dorsalis pedis artery, intact capillary refill for all 5 toes with good color and warmth, and mild swelling to the left knee. Computed tomography angiography noted a nonocclusive dissection flap to the left popliteal artery (Fig).

**Figure:**
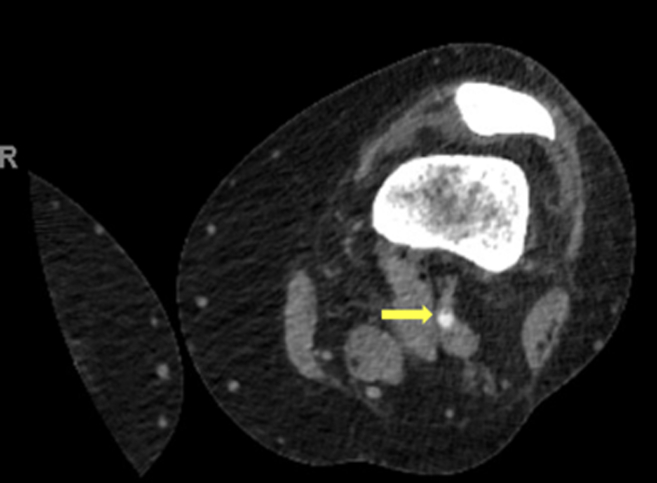
Computed tomography angiography with contrast demonstrates a nonobstructive dissection flap in the left popliteal artery at the level of the distal femoral metaphysis (arrow).

## 2 Diagnosis: Nonobstructive Dissection of The Left Popliteal Artery

The patient was admitted under observation for ongoing vascular examinations and had a left lower leg ultrasound arterial duplex without obstruction of flow. The patient was discharged home with vascular outpatient follow-up.

Geniculate artery embolization is a novel and minimally invasive intra-arterial procedure that selectively embolizes the geniculate branches as a treatment for pain in mild-moderate knee osteoarthritis.^1^^,^^2^ A catheter is inserted into a blood vessel, usually the femoral artery, and guided to the genicular arteries supplying the knee joint. This procedure has low rates of adverse events, which can include bone infarcts, fat necrosis, and temporary paresthesia.^3^^,^^4^ The popliteal artery is a major blood vessel in the lower leg, and the genicular arteries branch off from it to supply the knee joint. Dissection of peripheral arteries is rare but has occurred.^5^ Given the patient’s recent procedure, timing of symptoms, and imaging findings, the procedure likely caused the rare complication of a dissection flap.

## Funding and Support

By *JACEP Open* policy, all authors are required to disclose any and all commercial, financial, and other relationships in any way related to the subject of this article as per ICMJE conflict of interest guidelines (see www.icmje.org). The authors have stated that no such relationships exist.

## Conflict of Interest

All authors have affirmed they have no conflicts of interest to declare.
